# The perinucleolar compartment: structure, function, and utility in anti-cancer drug development

**DOI:** 10.1080/19491034.2024.2306777

**Published:** 2024-01-27

**Authors:** Eugene V. Makeyev, Sui Huang

**Affiliations:** a Centre for Developmental Neurobiology, King’s College London, London, UK; b Department of Cell and Developmental Biology, Northwestern University, Chicago, IL, USA

**Keywords:** Anti-cancer drug development, non-coding RNA, nucleolus, PNC, RNA binding proteins

## Abstract

The perinucleolar compartment (PNC) was initially identified as a nuclear structure enriched for the polypyrimidine tract-binding protein. Since then, the PNC has been implicated in carcinogenesis. The prevalence of this compartment is positively correlated with disease progression in various types of cancer, and its expression in primary tumors is linked to worse patient outcomes. Using the PNC as a surrogate marker for anti-cancer drug efficacy has led to the development of a clinical candidate for anti-metastasis therapies. The PNC is a multicomponent nuclear body situated at the periphery of the nucleolus. Thus far, several non-coding RNAs and RNA-binding proteins have been identified as the PNC components. Here, we summarize the current understanding of the structure and function of the PNC, as well as its recurrent links to cancer progression and metastasis.

## Introduction

The nucleus serves as a hub for genome organization and the regulation of gene expression [1–5]. It is indispensable for critical cellular functions, such as the replication of DNA and the production and processing of both coding and non-coding transcripts. Important physiological programs, including the regulation of the cell cycle, different types of stress response, and the maintenance of the overall cellular homeostasis, are to a large extent orchestrated in this part of the cell.

Despite the absence of membrane-enclosed organelles, the nucleus exhibits a high degree of spatial organization. Many nuclear activities are associated with their different non-membrane-enclosed domains [6]. For example, individual chromosomes are arranged into distinct territories rather than being randomly dispersed throughout the nucleus [7,8]. The nucleolus is a large nuclear body specializing in ribosome biogenesis and coordinating ribosomal DNA transcription and pre-rRNA processing and modification with the pre-ribosome assembly and other activities [9–15]. Many specialized nuclear domains, including Cajal bodies, Histone locus bodies, PML bodies, nuclear speckles, paraspeckles, etc., are identified by the expression of characteristic molecular markers [6]. The assembly of these membraneless structures is thought to rely on specific protein–protein, protein–RNA, protein–DNA, and protein–RNA–DNA interactions, as well as liquid–liquid phase separation driven by the physical and chemical properties of their constituents [6,16].

The perinucleolar compartment (PNC) is a nuclear body that forms under pathological conditions, specifically in cancer cells. Here, we summarize the current understanding of the PNC composition, and possible functions, along with its utility in the development of anti-cancer therapies.

## PNC discovery and initial characterization

**Figure 1.**
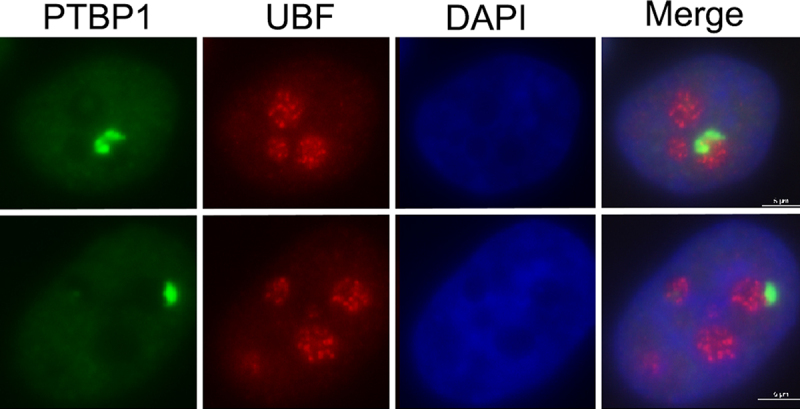
Immunofluorescence images showing PNCs detected by a PTBP1-specific antibody (green) and nucleoli labeled with an anti-UBF antibody (red). Nuclei are visualized by DAPI staining (blue) scale bar, 5 μm.

The discovery of the PNC traces back to the characterization of the polypyrimidine tract-binding protein (PTBP1). Immunolabeling of PTBP1 in HeLa cells revealed prominent microscopic structures located at the periphery of nucleoli [17]. Subsequently, Matera et al. identified an enrichment of non-coding RNAs including ribozymes in the structure, termed it ‘perinucleolar compartment’ [18,19]. While searching for cancer cell-specific nuclear markers in the mid-1990s, a monoclonal hybridoma screen was used to identify antibodies that specifically label nuclear structures in HeLa but not in NIH3T3 cells (our unpublished data). The screen yielded the SH54 antibody clone, which detects a nuclear body at the nucleolar periphery in HeLa cells but not in NIH3T3 cells. Further analyses showed that that SH54 recognizes PTBP1, and its immunolabeling patterns in HeLa cells are essentially identical to those described by Getti et al. [17,20] (Figure 1).

PNCs are irregular structures ranging from 1 to 2.5 μm in length and varying in width. They sometimes appear to be half-moon- shaped, following the contour of the nucleolus or invaginating into the nucleolus [20,21]. PNCs disassemble when the nucleolus breaks apart as cells enter mitosis and re-form along with the nucleolus in early G1 cells [20]. Thin-section (50 nm) transmission electron microscopic evaluation of optimally fixed HeLa cells showed that PNCs are electron-dense reticulated structures, morphologically distinct from the tripartite arrangement of the nucleolus. However, PNCs and nucleoli are structurally linked [20]. The physical connection between these two compartments was subsequently validated through the observation that a GFP-PTBP1 marked PNCs consistently co-purified with nucleoli (our unpublished studies) and was resistant to detergent extractions [21]. Importantly, the PNC is distinct from other known nuclear bodies [19,22] (and our unpublished data).

## Links with carcinogenesis

**Figure 2.**
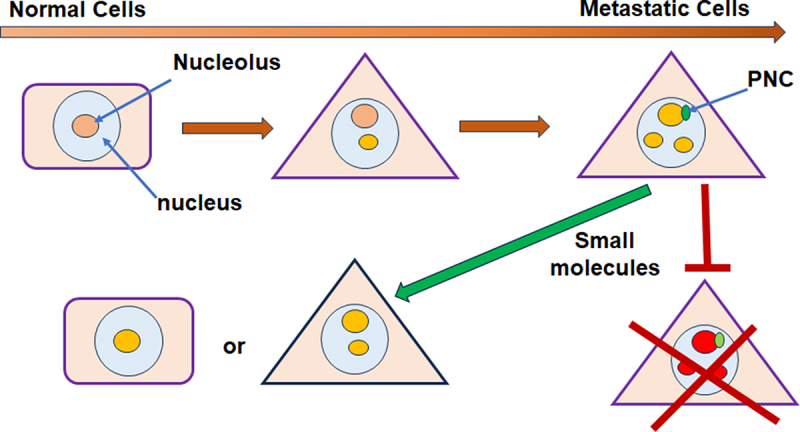
Using the PNC as a readout in metastasis-selective compound screens.

Quantification of PNC prevalence (i.e. the percentage of cells expressing at least one PNC) across a variety of cell lines demonstrated that PNCs are characteristic for cancer but absent in non-transformed cells, including embryonic stem cells [20,23]. PNCs are occasionally detected in some immortalized cells [23]. Notably, the prevalence of this nuclear body across different cancer cell lines varies from less than 5% to nearly 100%. Studies using a series of derivatives from a prostate cancer cell line PC3 with different metastatic properties [24] showed that PNC prevalence increases as a function of the metastatic potential [23]. Furthermore, the PNC is more prevalent in pancreatic cancer cell lines derived from the metastatic disease than those from more localized tumors [25]. Examination of patient samples from primary tumors revealed a positive correlation between PNC prevalence and the disease progression in breast, colorectal, and ovarian cancers [26,27] and reached highest in liver metastatic lesions [26]. Particularly, a high PNC expression in primary tumors of breast cancer patients associated with higher incidences of secondary diseases with distal metastases [26]. PNC prevalence is negatively correlated with patient outcomes in all three types of cancer examined [26,27]. Furthermore, detection of one of the non-coding RNA, PNCTR, revealed highly expressed PNCs in metastatic lymph nodes [28]. These observations suggest that the PNC forms as a result of complex, multi-step molecular events unfolding during cancer progression. The emergence of PNCs may be associated with significant changes that enable cancer cells to metastasize [29] (Figure 2).

## PNC as a surrogate marker for anti-cancer drug development

The strong correlation between the presence of PNCs and cancer metastasis, both in vitro and in vivo, underscores the potential of this compartment to serve as a surrogate marker for the malignant behavior of cancer cells [23,29]. Indeed, this multi-component cytological marker could more reliably reflect the highly heterogeneous and complicated nature of cancer metastasis than individual gene products, providing a valuable readout for the malignant potential within a given cell population. Building upon this hypothesis, a one-step high-content-put screening was developed, aiming to sift through structurally diverse chemical libraries to identify small molecules capable of reducing PNC prevalence [30]. The fundamental assumption behind this strategy was that diminishing PNC prevalence could stem from either interfering with cellular activities essential for maintaining the metastatic potential of cancer cells or selectively inhibiting cells that harbor PNCs.

The advantages of this approach were threefold. Firstly, the current ambiguity regarding the mechanism(s) of metastasis and the absence of any singular factor or pathway established as both necessary and sufficient for this process emphasize that the metastatic behavior of cancer cells may depend on a multitude of factors. With this in mind, we argued that using a complex structure such as the PNC as a marker might increase the likelihood of identifying small molecules with valuable ‘multitasking’ properties against multiple targets that are important for metastasis. Secondly, the presence of PNCs in cancer but not in normal cells offered an opportunity to identify compounds selectively targeting cancer cells. The selectivity could enhance therapeutic specificity and reduce nonspecific toxicity common in conventional chemotherapies. Finally, the detection of PNCs in cancer cells expressing the GFP-PTBP1 marker provided a robust one-step assay for high-content phenotypic screening of diverse compound libraries [30].

A high-content screen involving 140,000 compounds identified nearly 100 potential hits [30]. Secondary and tertiary assays eliminated compounds with nonspecific genotoxic, apoptotic, cytotoxic, and ATPase-inhibiting effects. The remaining hits were assessed for their capacity to inhibit Matrigel invasion. Among the top hits, one underwent significant modifications via medicinal chemistry, resulting in the development of a lead compound, metarrestin, which exhibited enhanced potency and favorable pharmacokinetic properties compared to the original hit [31].

Metarrestin effectively reduced PNC prevalence in treated cancer cell lines within 5 hours of treatment with the IC_50_ ranging between 100 and 300 nM for different cancer cell lines. Importantly, it demonstrated selective growth inhibition of a cancer over a normal cell line [25]. Metarrestin treatment of animals bearing orthotopic pancreatic cancer xenograft showed selective inhibition against distal metastasis to lung and liver, with little impact to the primary tumors. Survival studies revealed that administering metarrestin prior to the establishment of macro-metastases resulted in 100% survival after three months of treatment, while vehicle-treated animals succumbed to extensive metastasis. In addition, treatment after the formation of macro-metastases significantly extended survival up to four months. However, in this case, animals eventually succumbed, mainly due to the growth of primary tumors, with lungs and livers remaining relatively unaffected. Moreover, metarrestin exhibited efficacy in reducing metastasis in a prostate cancer xenograft model and in inhibiting the growth of a patient-derived xenograft originating from lung metastases of a breast cancer patient. These results collectively demonstrate that metarrestin selectively inhibits cancer metastasis without observable adverse effects on treated mice over several months.

The efficacy of metarrestin serves as proof of the concept that employing the PNC as a surrogate marker provides a productive strategy for identifying anti-metastatic drug candidates. While metarrestin is undergoing clinical trials, the next challenge is to go beyond the current use of the PNC as a marker and to understand the molecular mechanisms linking this nuclear compartment with cancer progression

## Insights into PNC structure

Since its initial description, a growing number of macromolecules have been found concentrated within the PNC. In the 1990s, Matera et al. demonstrated that non-coding RNAs, including RNA components of ribozymes, accumulated in PNCs within HeLa cells. Using in situ hybridization with antisense oligonucleotide probes, they showed that the Y1, Y4, and Y5 RNAs, as well as the RNA components of RNAses MRP and P colocalized with PTBP1 staining in the PNC [18,19]. Subsequently, other non-coding RNAs including Alu, RNA components of SRP particle, and PNCTR were also found enriched in the PNC [28,32]. Multiple proteins with RNA-binding properties have also been identified as PNC components. These include CUG-BP1, KSRP, Raver1 and 2, Rod1, nucleolin, and CDK13. At first glance, this list appears to be a random assortment of RNAs and proteins without clear relatedness. However, we believe that further analyses of this complex mixture, along with systematic identification of additional components, may provide important clues for the role that the PNC plays in cancer cells.

## PNC non-coding RNA components

An intriguing feature of the PNC is its association with diverse groups of non-coding RNAs. For example, of the four Ro-interacting Pol III transcripts examined by in situ hybridization (Y1, Y3, Y4 and Y5), three were detected in the PNC (Y1, Y4 and Y5) [19]. Consistent with the predominantly cytoplasmic localization of Ro RNPs, the PNC was not detected by staining HeLa cells with a Ro protein-specific antibody [19]. Furthermore, while the Ro RNP could be readily extracted from the cytoplasm, the Y1, Y4, and Y5 PNC signals remained detectable under the same treatment conditions [19]. Similarly, the RNA components of RNAse MRP (RMRP) and RNAse P (RPPH1), also synthesized by Pol III, were also localized to the PNCs [18,19,32]. However, the attempts to detect a protein component of the ribozymes in PNCs did not yield any signals [33]. Sucrose gradient experiments showed that RMRP was co-fractionated with PTBP1 and CUGBP1 in larger complexes compared to the ribozymes. The protein–RNA interaction was confirmed through co-precipitation in those specific fractions [33]. Taken together, these findings support the idea that the Ro and ribozyme RNAs assemble distinct complexes inside and outside of the PNC.

In 2018, Yap et al. reported the presence of a highly concentrated long non-coding RNA called PNCTR within the PNC [28]. PNCTR is a sizable RNA, spanning over 10 kilobases, and is rich in (UC)n simple repeat sequences that bind multiple copies of PTBP1. PNCTR is transcribed by RNA polymerase I and is expressed at elevated levels in cancer cells. Interestingly, PNCs enriched with PNCTR were detected in metastatic lesions within lymph nodes [28]. PNCTR knockdown reduced PNC prevalence and inhibited cell growth by triggering apoptosis. Reduction of PNCTR that also changed splicing patterns is a subset of pre-mRNA targets, with some of these events regulated by PTBP1 directly. The current model is that the partial sequestration of PTBP1 in the PNC limits its ability to interact with splicing targets in the nucleoplasm. As PTBP1 has been reported to have pro-apoptotic activities, such sequestration mechanism may also help cancer cells to avoid programed cell death [28].

## PNC protein components

RNAs localized within PNCs have an affinity for binding PTBP1, and in some cases, CUGBP1. PTBP1 is known for its interaction with polypyrimidine-rich RNAs and plays a crucial role in various aspects of RNA metabolism, including polyadenylation, mRNA stability, pre-mRNA splicing, alternative splicing, and translational regulation of mRNAs [34–36]. PTBP1, but not CUGBP1, knockdown disrupts the PNC integrity, underscoring its central structural role [32]. Additionally, Raver1 [37,38], Raver2 [38], nPTB/PTBP2 and Rod1/PTBP3 (our unpublished data), which share homology with PTBP1 and possess similar RNA-binding motifs, also interact with PTBP1. Similar to PTBP1, these proteins are involved in the regulation of pre-mRNA splicing, in addition to their other cellular functions.

CDK13, a cyclin-dependent serine-threonine kinase, is a somewhat unusual PNC component. The CDK family is well-known for its contribution to the cell cycle control [39]. CDK13 has been shown to interact with splicing regulators [40] and localize to the nuclear speckles enriched in pre-mRNA splicing factors in the nucleus, in addition to its PNC localization [22]. CDK13 is important for the PNC structure, as a knockdown of the protein reduced PNC prevalence [22]. Recently, CDK13 has been shown to play important roles in damaged RNA surveillance [41,42]. Mutations in CDK13 associate with more aggressive forms of melanoma, suggesting that the removal of impaired RNA serves to deter tumorigenesis [42,43]. The well-defined cell cycle-related substrates of CDK13 were not found in the PNC [22], suggesting PNC-localized CDK13 does not form its known functional complexes. It is therefore possible that the PNC sequesters CDK13 away from its normal function in cell cycle regulation and in damaged RNA surveillance, thus promoting carcinogenesis.

## PNC assembly in cancer cells

We now know that the PNC contains a repetitive long-noncoding RNA, the RNA components of ribozymes, SRP, and Ro RNPs. It also harbors proteins involved in diverse processes from RNA stability and splicing to cell cycle progression and damaged RNA surveillance. Our recent analysis of PNC-associated proteome using a proximity labeling approach identified an extended list of protein candidates [44], which will be interesting to follow up on in further functional studies.

The idea of sequestering PTBP1 [28] or CDK13 [22] by highly repetitive RNA such as PNCTR or Alu to prevent them from normal functions might connect the PNC assembly process with its functions in high-grade and metastatic cancer cells. For example, CDK13 is considered a tumor suppressor and plays a crucial role in maintaining cellular integrity [42]. Raver1 plays an important role in modulating focal adhesion and cell–cell interactions [45], and its sequestration could change the cell junction dynamics favoring a metastatic behavior.

The significance of the PNC enrichment of apparently nonfunctional Pol III RNA products [19,33] is currently unclear. Earlier studies showed that PNCs are heavily incorporated with BrU after a brief (5 minutes) pulse labeling, suggesting it is a either a site of transcription or a transit depot for newly synthesized RNA [21]. In situ hybridization analyses of Pol III-dependent genes encoding Y1, Y4 [19], and RMRP [32] showed that these loci did not colocalize with the PNCs. A high-resolution light microscopy assessment of PNC structures revealed that RMRP predominantly colocalized with PTBP1, rather than with the BrU foci [32]. Thus, PNC is not the sites of the transcription for these Pol III RNAs and they must be transported to the PNCs post-transcriptionally.

The inhibition of Pol I or Pol III transcription disrupts the PNC structure [21,25,32]. Pol I may contribute to the PNCs integrity in two possible ways. As PNCTR is a Pol I transcript whose level is important for PNC structure [28], inhibiting Pol I can disrupt the PNC by dampening PNCTR levels. Furthermore, such treatments can disrupt the nucleolus, to which the PNC is physically linked. Indeed, knockdown of a Pol I subunit induced nucleolar segregation and reduced PNC prevalence [46]. Since Pol I transcription factors are not detectable in the PNC (Figure 1), it remains to be seen whether the PNC is a site of active Pol I transcription or if PNCTR is transported to the PNC, similar to the Pol III transcripts. The PNC integrity was also compromised in response to Pol III inhibitor treatments, highlighting the importance of Pol III transcripts delivered to this nuclear body from other nuclear locations.

An exciting question for the future is how the site of PNC assembly is specified. PNCs are generally heritable across cell divisions. Disruption of the PNC by genotoxic agents, particularly topoisomerase I and II inhibitors, underscores its intimate connection with DNA and chromatin . In addition, in a temperature-sensitive mutant where endoreplication occured at a non-permissive temperature, a direct correlation was observed between the frequency of endoreplication cycles and the abundance of PNCs per cell [47]. Efforts are currently underway to identify the chromatin domains physically associated with the PNC. Perhaps the transcription of these yet-to-be-identified loci will explain the robust incorporation of BrU in pulse labeling experiments and shed light on the mechanisms orchestrating the PNC assembly in cancer cells.

## Summary

**Figure 3.**
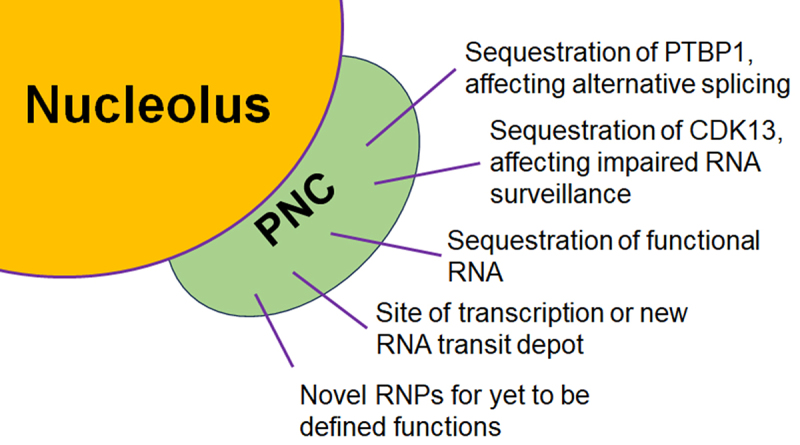
Possible PNC functions in cancer cells.

The PNC emerges in cancer cells as a complex assembly of non-coding RNAs and RNA-binding proteins, which might be associated with specific chromatin sites. Interestingly, several Pol III-dependent RNA components localizing to the PNC do not conform to their canonical RNP complexes, located elsewhere in the cell. Another non-coding RNA critical for PNC structure and function is the Pol I transcript PNCTR. The PNC also hosts various protein regulators of cellular RNA metabolism. These factors do not necessarily form their conventional functional complexes within this compartment. It is possible that repetitive RNAs sequester proteins like PTBP1 or CDK13, thus hindering their normal functions and promoting carcinogenesis. Intriguingly, RMRP is detected in a large protein complex with PTBP1 and CUGBP2 in PNCs, spatially separated from the newly synthesized RNA. Are such RNPs involved in transcriptional regulation of PNC-associated genes? Does PNC also sequester RNAs, preventing them from their normal functions (Figure 3)? Further research is needed to answer these and other questions pertaining to PNC biology and to improve our understanding of the functional links between this nuclear compartment and cancer progression and metastasis. At the meantime, PNCs as a surrogate marker for cancer metastasis have been used to develop selective anti-cancer therapeutics.
